# Why Can’t I Stop Smoking: Predictors of Tobacco Use and Quit Rates in the Freedom From Tobacco Program

**DOI:** 10.7759/cureus.41649

**Published:** 2023-07-10

**Authors:** Regina G Moore Ude, R. Patti Herring, Mohamed Ismail, Keiji Oda, Khaled Bahjri, Wenes P Reis, Josileide Gaio, Hildemar Dos Santos

**Affiliations:** 1 Health and Human Ecology, California State University, San Bernardino, USA; 2 School of Public Heath, Loma Linda University Medical Center, Loma Linda, USA; 3 Internal Medicine, Kaiser Permanente, Riverside, USA; 4 School of Public Health, Loma Linda University Medical Center, Loma Linda, USA; 5 Clinical Research, New World Medical, Rancho Cucamonga, USA; 6 Public Health, Loma Linda University Medical Center, Loma Linda, USA; 7 Preventive Care, Loma Linda University School of Public Health, Loma Linda, USA

**Keywords:** southern california permanente medical group, social-ecological model, freedom from tobacco program, smoking cessation, smoking

## Abstract

Introduction

As the leading cause of preventable chronic diseases in adults 18 years and older, tobacco usage in the U.S. results in over 20 million premature deaths annually. Current smokers might need extra support on the path to successfully quitting.

Aim

To evaluate the influence of predictors of smoking-on-smoking cessation in the Freedom From Tobacco Program (FFT) offered by Southern California Permanente Medical Group (SCPMG).

Methods

This was a quasi-experimental study to evaluate rates of smoking cessation among participants in the FFT program. There were 471 participants in the study. Factors of the Social Ecological Model (SEM) and demographics were examined to determine if they could predict tobacco cessation. The SEM suggests that an individual’s behavior is integrated into a network of intrapersonal characteristics, interpersonal processes, institutional factors, community features, and public policy. In particular, the study mainly addressed the institutional factor. It was promoted within a Health Management Organization and the interpersonal process because it was a group intervention.

Findings

After multiple regression analyses with all predictors from the SEM and demographics, the only significant predictor was the number of previous attempts to quit. Smokers who tried to stop four or more times in the past were 2.6 times (p<0.03) more likely to quit than those who tried fewer times. As we are aware, this was the first time this result was found for programs implemented by Health Management Organizations. The general quit rate at 12 months for the FFT program was 43.1%.

Conclusion

As the only predictor of quitting in this study was the number of previous attempts to quit smoking, the recommendation is to develop longer-term smoking cessation programs or a longer follow-up to facilitate smokers who relapse to go back and try to quit again. Another recommendation is to identify the main reasons for relapse and try to address these factors in further interventions.

## Introduction

Tobacco use accounts for over 450,000 premature deaths in the United States [1]. In the last 50 years, more than 20 million Americans have died as a result of tobacco use. In addition, parental smoking has caused over 100,000 infant deaths; at this rate, 5.6 million children under the age of 18 alive today will die prematurely from a smoking-related illness [2]. 

The primary objective of this study was to evaluate the 12-month quit statuses of participants who attended the Freedom From Tobacco Program (FFT) class(es) at Southern California Permanente medical centers and examine associations with predictors of tobacco use. To examine the associations, a behavioral change theory was utilized. Behavior change theories aid in the understanding of why people choose to use tobacco products, knowing the harmful effects, and why they continue to do so when they know they should not [3,4]. The behavioral theory guiding this study is the Social-Ecological Model (SEM). 

Urie Bronfenbrenner formalized the SEM to explain the interconnectedness and evolving association between society, community, interpersonal relationships, and individuals [5]. The SEM suggests that an individual’s behavior is integrated into a network of intrapersonal characteristics, interpersonal processes, institutional factors, community features, and public policy [6]. These may include individual influences such as smoking to relieve stress as well as influences from family, social networks, the organization's people, the community where they work, live, and play, and the society in which they live, which often shape human behavior and the way humans respond to behavior change [7]. The SEM includes the following levels: individual/intrapersonal, interpersonal, community, organizational and policy/enabling environment, physical environment, and culture. This study used an adapted SEM that included four levels of the model; individual/intrapersonal; interpersonal, organizational, and policy/enabling/environment. 

The intrapersonal/individual level examined the smokers’ knowledge (i.e., knowing the health effects of smoking and attempting to quit), attitude, belief, and awareness of the health risks that influence behavior [6]. 

The interpersonal level examines both formal and informal social networks and supports systems that can influence a smoker’s behavior. These networks/support systems may include healthcare providers, family, friends, peers, and co-workers [6]. 

The organizational level examines organizational or social institutions’ rules and regulations, policies, and informal structures that may constrain or promote recommended behaviors [8]. 

The last level, the policy/enabling environment level, examines local, state, and federal policies that regulate or support healthy actions and practices for disease prevention, early detection, control, and management [8].

This study aims to determine which factors of the social-ecological model (i.e., individual, interpersonal, organizational, and environmental) assessed at baseline influenced smoking cessation rates, controlling for demographics such as age, gender, marital status, education, and ethnicity.

## Materials and methods

Participants enrolled in the Southern California Permanente Medical Group (SCPMG) Freedom From Tobacco program, which was a behavior change program facilitated by a certified health educator. The seven-week in-person FFT classes were aimed at priming members to gain independence from tobacco use by encouraging them to establish quit dates and preparing their surroundings for a smoke-free environment. The classes addressed the key factors of tobacco addiction, including the habitual (i.e., identifying triggers), psychological (i.e., dealing with stress), and medical components of tobacco use. The classes were offered free to members at each of the 12 Southern California Permanente Medical Group Medical Centers. Members could also secure Food and Drug Administration (FDA)-approved tobacco dependence treatment medication at a standard co-payment if desired [9]. 

**Figure 1:**
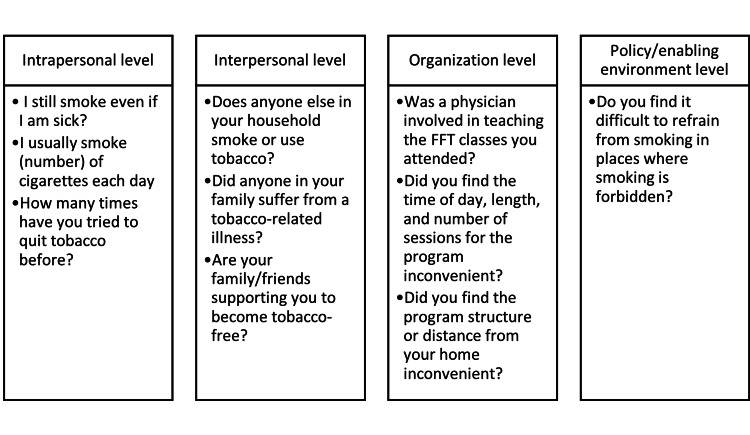
Questions examined for each of the SEM levels

A total of 1015 participants attended FFT classes. Smoking status at 12 months was confirmed for a total of 471 participants in the study. Smoking status couldn’t be obtained from the 544 participants who were dropped from the study. Five participants were excluded from the study because they were over the age of 87. During the orientation, participants were asked to self-report data regarding their medical health, lifestyle behaviors, and smoking cessation readiness at each of the SEM levels (Figure 1).

This study was approved by the Loma Linda University committee under IRB number 5190100. 

Statistical analysis

For the statistical analysis, we used IBM Corp. Released 2017. IBM SPSS Statistics for Windows, Version 25.0. Armonk, NY: IBM Corp. to run an analysis of the association between predictors of tobacco use. We then performed a bivariate analysis to access the factors associated with quitting smoking after 12 months using the chi-square methodology. 

## Results

Table 1 shows the description of the participants regarding demographics and predictors of smoking.

**Table 1:** Freedom From Tobacco Program study participants

		N	%
Age	40 or less	41	8.9%
	More than 40	422	91.1%
Gender	Male	253	53.7%
	Female	218	46.3%
Education	High School	183	44%
	College	233	56%
Race/Ethnicity	Whites	265	59.4%
	Minorities	181	40.6%
Marital Status	Married	282	62.7%
	Singles	168	37.3%
Difficulty to Refrain From Smoking in Forbidden Places	No	365	77.5%
	Yes	106	22.5%
Physician Involvement	No	428	90.9%
	Yes	43	9.1%
Tobacco Related Family Illness	No	324	68.8%
	Yes	147	31.2%
Family/Friends Support	No	61	12.9%
	Yes	410	87.1%
Other Household Smokers	No	322	68.4%
	Yes	149	31.6%
Number of Quit Attempts	Never	37	8.2%
	1-3	282	62.1%
	4 or more	135	29.7%
	Number of Cigarettes Smoked Per Day	10 or Less	126	26.7%
11 to 20		219	46.5%
More than 20		126	26.8%
Smoke While Sick	No	231	49.1%
	Yes	240	50.9%
Program Inconvenience Time of Day	No	458	97.2%
	Yes	13	2.8%
Program Inconvenience Total Number of Sessions	No	469	99.6%
	Yes	2	0.4%
Program Inconvenience Length of Each Session	No	469	99.6%
	Yes	2	0.4%
Program Inconvenience Program Structure/Format	No	469	99.6%
	Yes	2	0.4%
Program Inconvenience Distance From My Home	No	452	96%
	Yes	19	4%

The main question for this study was, “What factors of the social-ecological model (i.e., individual, interpersonal, organizational, and environmental) influenced the smoking cessation status of the participants of the FFT classes? There were no statistically significant differences in quitting rates according to participants' demographics (age, gender, education, race, and marital status).

At first, when we conducted individual analyses (bivariates) comparing variables with each other, only two predictors were significant: one was from the interpersonal level of the social-ecological model, specifically having ‘tobacco-related family illness” (p=0.05) and was inversely related to quitting smoking. The other was from the intrapersonal/individual level; those who smoked when sick (P= 0.04) were more likely to stop smoking at 12 months (Table 2). 

**Table 2:** Association between SEM factors and smoking quitting status at 12-months

		Quit		Smoke		
		N	%	N	%	P-value
Age	40 or less	26	63.4%	15	36.6%	0.371
	More than 40	237	56.2%	185	43.8%	
Gender	Male	135	53.4%	118	46.6%	0.116
	Female	132	60.6%	86	39.4%	
Education	High School	105	57.4%	78	42.6%	0.952
	College	133	57.1%	100	42.9%	
Race/Ethnicity	Whites	145	54.7%	120	45.3%	0.204
	Minorities	110	60.8%	71	39.2%	
Marital Status	Married	156	55.3%	126	44.7%	0.455
	Singles	99	58.9%	69	41.1%	
Difficulty to Refrain From Smoking in Forbidden Places	No	203	55.6%	162	44.4%	0.384
	Yes	64	60.4%	42	39.6%	
Physician Involvement	No	240	56.1%	188	43.9%	0.297
	Yes	27	62.8%	16	37.2%	
Tobacco Related Family Illness	No	174	53.7%	150	46.3%	0.052**
	Yes	93	63.3%	54	36.7%	
Family/Friends Support	No	35	57.4%	26	42.6%	0.907
	Yes	232	56.6%	178	43.4%	
Other Household Smokers	No	179	55.6%	143	44.4%	0.480
	Yes	88	59.1%	61	40.9%	
Number of Quit Attempts	Never	23	62.2%	14	37.8%	0.138
	1-3	169	59.9%	113	40.1%	
	4 or more	68	50.4%	67	49.6%	
Number of Cigarettes Smoked Per Day	10 or Less	74	58.7%	52	41.3%	0.858
	11 to 20	122	55.7%	97	44.3%	
	More than 20	71	56.3%	55	43.7%	
Smoke While Sick	No	120	51.9%	111	48.1%	0.042*
	Yes	147	61.3%	93	38.8%	
Program Inconvenience Time of Day	No	258	56.3%	200	43.7%	0.355
	Yes	9	69.2%	4	30.8%	
Program Inconvenience Total Number of Sessions	No	265	56.5%	204	43.5%	0.508
	Yes	2	100.0%	0	0.0%	
Program Inconvenience Length of Each Session	No	265	56.5%	204	43.5%	0.508
	Yes	2	100.0%	0	0.0%	
Program Inconvenience Program Structure/Format	No	266	56.7%	203	43.3%	>0.999
	Yes	1	50.0%	1	50.0%	
Program Inconvenience Distance From My Home	No	254	56.2%	198	43.8%	0.292
	Yes	13	68.4%	6	31.6%	

However, when we designed a multivariate logistic regression model (Table 3), including all variables in the study (predictors and demographics), the two previous factors found in the bivariates did not maintain their significance. But another variable emerged as significant in this last model. The results indicated that smokers who previously attempted to quit four or more times (intrapersonal) were 2.6 times more likely to quit smoking than smokers who never made a quit attempt (p=0.03). The ones who tried to quit only 1-3 times did not relate to quitting status (p=0.22), but the trend was in the same direction. Finally, the quit rate for the whole sample at 12 months after the end of the program was 43.1%.

**Table 3:** Multivariate logistic regression on predictors of quitting smoking at 12-months after adjusting for demographic factors * indicates significance at an alpha of 0.05

	B	S.E.	P-value	Odds Ratio	95% Lower	95% Upper
Age (40 or Less vs. More Than 40)	-.325	0.395	0.411	0.723	0.333	1.569
Gender (Male vs. Female)	0.264	0.234	0.261	1.302	0.822	2.061
Education (High School vs. College)	0.057	0.218	0.792	1.059	0.691	1.622
Race (Whites vs. Minorities)	0.163	0.236	0.491	1.177	0.740	1.870
Marital Status (Married vs. Singles)	0.097	0.230	0.672	1.102	0.702	1.730
Diff. Refrain. From Smoking in Forbidden Places (Yes vs. No)	-.310	0.276	0.261	0.734	0.427	1.259
Physician Involvement (Yes vs. No)	-.417	0.374	0.265	0.659	0.317	1.371
Tobacco Related Family Illness (Yes vs. No)	-.444	0.238	0.062	0.641	0.402	1.023
Family/Friends Support (Yes vs. No)	0.255	0.372	0.494	1.290	0.622	2.674
Nos. of Quit Attempts (1-3 vs. Never)	0.535	0.436	0.220	1.708	0.700	4.013
Nos. of Quit Attempts (4 or More vs. Never)	0.974	0.457	0.033*	2.649	1.082	6.481
Nos. of Cigarettes Smoked Per Day (11 to 20 vs. 10 or less)	0.101	0.279	0.717	1.106	0.640	1.912
Nos. of Cigarettes Smoked Per Day (More than 20 vs. 10 or less)	0.218	0.332	0.510	1.244	0.650	2.382
Smoke While Sick (Yes vs. No)	-0.316	0.230	0.168	0.729	0.465	1.143
Other Household Smokers (Yes vs. No)	-0.075	0.229	0.742	0.927	0.592	1.452
Prog. Inconvenience Time of Day (Yes vs. No)	-0.817	0.822	0.321	0.442	0.088	2.215
Prog. Inconvenience Length of each Session (Yes vs. No)	-21.094	28245.74	0.999	-	-	
Prog. Inconvenience. Program Structure/Format (Yes vs. No)	-21.112	40192.970	1.000	-	-	
Prog. Inconvenience Distance From My Home (Yes vs. No)	-0.333	0.540	0.537	0.716	0.249	2.064

## Discussion

In our final model, only one variable was significant: the number of times a person tried to quit smoking in the past (Table 3). Those who had tried more than four times compared to never were 2.6 times more likely to quit (Table 3). This shows that in this study, only one characteristic of the social-ecological model was found to be significant: the intrapersonal level, specifically, the number of quit attempts in the past.

This is in accordance with the literature, as the American Cancer Society and the CDC both suggest 8-11 attempts before quitting permanently [10]. On average, most smokers attempt to quit smoking four or five times before cessation is successful [11]. However, study results suggest that smokers’ intentions to quit may differ depending on their experiences during their last failed quit attempt [12].

These results, even though confirmed by the literature in general, might be the first observed among participants of programs run by a health maintenance organization (HMO). We were not able to find any other HMO smoking cessation program with these results published in the literature. These unique results confirmed that even for programs targeting people with equal access to medical care, there is still a need for longer-term follow-up and multiple activities for smoking cessation. For instance, addressing the interpersonal level with the presence of former smokers in the sessions. 

Social-ecological model: interpersonal and intrapersonal factors

At the bivariate level, we found two factors that were significantly related to quitting rates: ‘tobacco-related family illness’ and ‘smoking while sick’. These are items of the Social-Ecological Model that focus on interpersonal and intrapersonal levels, respectively. 

The social-ecological perspective focuses on individual-level influences such as peer and family members' attitudes and behaviors [13]. The SEM recognizes that individuals live within multilayered social, physical, and cultural environments, and it is the interaction between the individual and their environments that underlie health outcomes [14].

In this study, we found that having a disease related to tobacco in the family did not lead to smoking cessation behavior. Those who have family illnesses were less likely to quit, at least during the first phase of our analysis. In our final analysis, with a multivariable test performed, significance was almost achieved, resulting in a p-value of 0.062. In the literature, there is enough evidence that fear itself is not enough motivation for people to change their behavior [15]. Unfortunately, awareness of the ill effects of tobacco use and access to healthcare most often occur in well-educated populations, which is why community-based interventions are vital to reaching the entire community [16].

At the intrapersonal or individual level, one important factor is the level of addiction to nicotine. If smokers are heavily addicted, they will have more difficulty quitting. In our study, during the bivariate analyses, we found that those who are probably heavy smokers who would still be smoking while they are sick are less likely to quit (Table 2). This is in agreement with the literature, as hard nicotine addiction is similar to other drug addictions and very hard to quit [17]. However, our final results did not confirm this finding, telling us that in this setting, there was no difference between this intrapersonal factor and quitting rates (Table 3).

Discussion about other intrapersonal and interpersonal factors of the SEM

Number of Cigarettes Smoked

Another intrapersonal factor evaluated by this study was the number of cigarettes smoked. In general, heavy smokers are less likely to quit smoking even during pregnancy and are more likely to relapse relative to light smokers [18]. Nondaily and light daily smokers are believed to benefit from cessation programs as well as have a higher prevalence of past attempts compared to daily smokers [19]. In our study, participants who smoked between 1 and 20 cigarettes per day had higher quit rates than those who smoked more than 20 cigarettes per day, but these results were not statistically different. 

Healthcare Provider’s Advice

Healthcare providers, especially physicians, have long been considered essential for cessation advice and assistance in abstinence rate reduction [20]. In one study, smokers expected their physicians to take an active role in their smoking cessation, but often these expectations were not met [21]. Expectations included discussing smoking cessation during the doctor’s visit, tailoring cessation approaches, providing information on the harm of tobacco smoking, and prescribing pharmacological or other cessation treatment. 

Surprisingly, discussion regarding tobacco use occurs less often in the doctor’s office than in testing for blood pressure, cholesterol, prostate, or breast cancer, although evidence suggests that a physician’s advice to quit smoking has a significant effect on quit success [22]. Again, our study did not find this to be significant, but we only measured the influence of the provider or medical doctor. It could be that if we investigated the whole team of health professionals involved in the program, we could probably find some significant results, as most of the interaction in the program was with the nurses and health educators rather than the doctors.

Other Household Smokers

A smoker’s immediate social environment plays an important role in their ability to sustain smoking cessation [23]. It is estimated that less than 50% of smokers will fail at permanent cessation, and living with other smokers in the household makes failed attempts more likely, often leading to relapse one year after beginning any cessation program [24]. Daily contact with other smokers reduces the likelihood of quitting success [25]. Family and friends' support can determine a smoker’s likelihood of attempting to quit and sustaining success [26].

In our study, other household smokers were not found to be significant, nor was the influence of friends and family on quitting rates (Table 2). This could be because, during the sessions, there was enough information and support for quitting smoking to overcome the potential negative influence of friends and family.

Organization/policy level

At the organizational level, institutions such as churches or work sites develop policies that prohibit smoking in and on their property. Within this level, programs aimed at restricting smoking at the workplace and stakeholder participation in program development targeted at the community level have shown success in some settings [27]. Policy-driven interventions aimed at promoting smoke-free environments, limiting access, and increasing tobacco prices through excise taxes have also assisted in decreasing the prevalence rate of tobacco use [27].

Smoking restrictions are associated with reduced smoking; however, there are gaps in the literature regarding the relationship between smoke-free policies and cessation [28]. In this population, our study also did not find any significant relationship. This may be because SCPMG members have been accustomed to not smoking on the hospital campus for years, so these restrictions would not make any difference to them. Another reason would be because our participants did not belong or did not work for an organization that has those policies. They were only members of a health management organization, the SCPMG, that promotes smoking cessation activities.

Probably, this study was not able to find relationships with other factors of the Social-Ecological Model because the questions were taken from a ready-made questionnaire to fulfill the theory. Ideally, questions designed by the theory founders, when designed previously for studies, would give more positive results.

Although the SEM has been effective in reducing the overall prevalence of tobacco use in the United States [7], only one factor of the model was found to be effective in reducing the rates of tobacco use among the participants in the FFT project. The fact that smokers who tried to quit more than four times in the past were more likely to quit confirms the literature on the subject and gives more support for developing comprehensive programs and providing adequate follow-up for participants.

Freedom from tobacco quitting rates

The general quit rate at 12 months for the FFT program was 43.1%. This result was published and discussed in another paper. However, it is important to report here that the program results in terms of smoking cessation at one year were high compared to the literature [29]. Also, this quitting rate could reflect the structure of the program, which included stop-smoking medication mostly for free (including free nicotine patches), strong medical support, the possibility to re-enter the smoking cessation classes, an online support website, and a smoking support hotline. All of these activities might be enough to counteract the predictors of smoking that were reported before the initiation of the classes and are potentially responsible for the lack of significant results in some areas of the SEM.

As the only predictor of stopping smoking in this study was the number of previous attempts to quit smoking, this result might be the first in the literature to show that members of a health management organization also have the same quitting behavior as other stop-smoking programs conducted in different settings [25,30]. This calls for the recommendation from the researchers that programs for smoking cessation should be structured for a longer period or with constant follow-ups, probably up to one year. A long-term follow-up would allow smokers to have more opportunities to try to stop smoking and become successful with fewer attempts. In any case, the quitting results of the program still show good quitting rates in general (41.3%), which could serve as an example for other health management organizations or other health care plans to be more proactive and implement more structured smoking cessation programs.

This study has some limitations. Questions regarding participants' medical health, lifestyle behaviors, and smoking cessation readiness at each of the SEM levels were all self-reported, which could threaten reliability and validity. Also, this is a specific population; for example, all have health insurance plans, and the results cannot be generalized to other populations.

## Conclusions

This study evaluated rates of smoking cessation among participants in the FFT program. There were 471 participants in the study, and after multiple regression analyses with all predictors from the SEM and demographics, the only significant predictor was the number of previous attempts to quit. Smokers who tried to stop four or more times in the past were 2.6 times (p<0.03) more likely to quit than those who tried fewer times. The recommendation is to develop longer-term smoking cessation programs or a longer follow-up to facilitate smokers who relapse to go back and try to quit again.
